# Advances in Electrocardiogram-Based Artificial Intelligence Reveal Multisystem Biomarkers

**Published:** 2025-03-24

**Authors:** Xichong Liu, Sabyasachi Bandyopadhyay, Albert J. Rogers

**Affiliations:** Department of Cardiology, Cardiovascular Institute, Stanford University School of Medicine, Stanford, USA

**Keywords:** Electrocardiogram (ECG), Deep learning, Health disparities, Artificial intelligence

## Abstract

As Artificial Intelligence (AI) plays an increasingly prominent role in society, its application in clinical cardiology is gaining traction by providing innovative diagnostic, prognostic, and therapeutic solutions. Electrocardiogram (ECG), as a ubiquitous diagnostic tool in cardiology, has emerged as the leading data source for Deep Learning (DL) applications. A recent study from our group used ECG-based DL model to identify cardiac wall motion abnormalities and outperformed expert human interpretation. Motivated by this work and that of many others, we aim to discuss advances, limitations, future directions, and equity considerations in DL models for ECG-based AI applications.

## INTRODUCTION

Electrocardiogram (ECG) is a widely available and accessible point-of-care diagnostic tool in cardiovascular medicine. Historically, ECGs provided critical diagnostic information to the bedside clinician, most commonly related to cardiac rhythm and myocardial ischemia. However, detection of structural cardiac abnormalities was limited and the utility of ECGs as a diagnostic tool for non-cardiac indications was low. In recent years, the advancement in Artificial Intelligence (AI) and Deep Learning (DL) techniques have successfully developed tools that provide invaluable diagnostic information for both cardiac and non-cardiac indications [1]. It is increasingly recognized that latent signals from ECGs beyond human interpretation can be readily extracted by AI models. With the rising prevalence of wearable consumer devices with ECG capabilities, there is growing interest to extend the diagnostic value of ECG beyond cardiovascular indications [2].

Despite the growing momentum for ECG-based AI diagnostics, the underrepresentation of diverse patient populations in cardiovascular research datasets is being increasingly recognized. This underrepresentation creates intrinsic bias in the prediction models and can lead to disparities in diagnostic performance [3]. Additionally, access to medical care and expensive consumer products may similarly skew models trained on data sets collected from wearable technologies, which poses further challenges to the development of equitable diagnostic tools for consumer products [2].

In this review, we aim to review recent progress in DL for ECG-based assessments of health across multiple organ systems, provide perspectives on barriers to their implementation, and discuss future directions for AI-enhanced research in cardiovascular health and beyond.

## LITERATURE REVIEW

### Overview of deep learning in ECG-based applications

**Figure 1:**
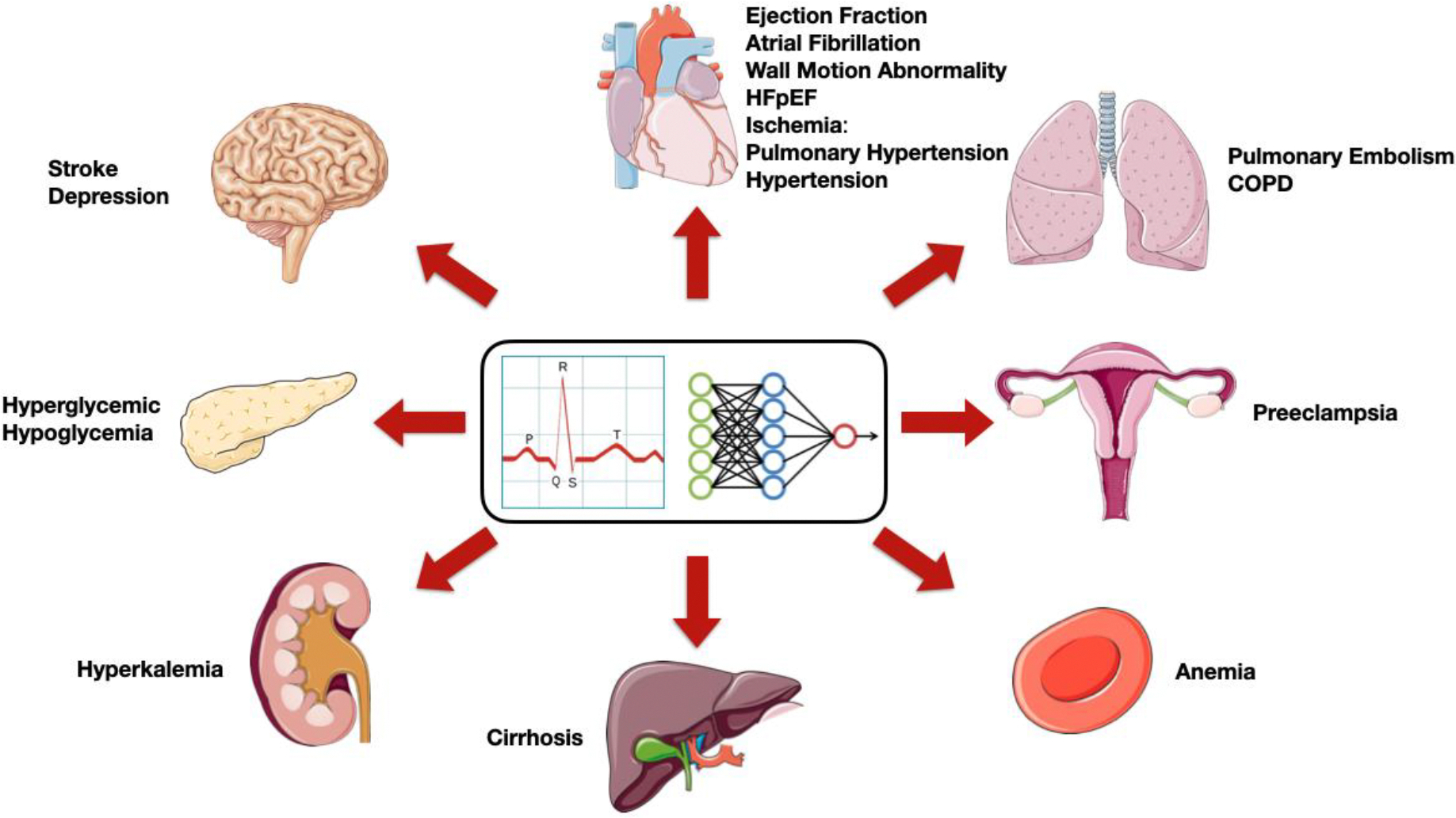
Illustration of cardiac and non-cardiac biomarkers for which DL ECG analysis has been applied.

DL has revolutionized ECG analysis through model architectures such as Convolutional Neural Networks (CNNs), Recurrent Neural Networks (RNNs), and hybrid models that integrate spatiotemporal feature extraction [1,4]. Model creation typically involves construction of training, testing and validation data sets by exporting ECGs and labeling using multimodal sources (laboratories, imaging, clinical diagnosis, etc.) to explore biomarkers related to the heart and other organs (Figure 1). The ECGs must be pre-processed to reduce background noise and baseline variations. The training sets are created using data from a single or several institutions, while testing is conducted on a holdout set of patients not previously seen by the model.

External validation on an external data set provides evidence of generalizability. For rarer conditions with limited data, federated learning across multiple institutions without data sharing have been proposed [5].

### Deep learning of the ECG for cardiac biomarkers

Numerous DL models of ECG have been developed for cardiac indications pertaining to functional characteristics, risk prediction, and structural information [5–10]. For instance, a recent study from our group showed that wall motion abnormality is identified with DL and outperformed both expert ECG interpretation and Q-wave indices, suggesting that additional physiologic signals related to ventricular function are captured by the model [7]. Similar work in acute myocardial ischemia also showed superior performance of the model compared to expert clinicians [9]. The prognostic value of DL models provide insights beyond the traditional score-based paradigm. Yuan et al,. developed a model that predicts risk of atrial fibrillation while in sinus rhythm, while Lee et al,. used ECG to identify patients with increased filling pressure and found a higher associated mortality rate [8,11].

### Deep learning of the ECG for non-cardiac biomarkers

While it is widely understood that a variety of physiological information is encoded in the 12-lead ECG, the diagnostic utility of traditional ECG in non-cardiac indications is limited when interpreted by human experts. With the hypothesis that ECG contains higher-dimensional spatiotemporal information beyond human interpretation, a variety of models have been developed to study disease entities with or without relationship to the cardiovascular system. Butler et al,. hypothesized that early cardiac involvement in women at risk for preeclampsia can be identified *via* DL ECG models and successfully showed high predictive accuracy for later development of preeclampsia [12]. Others have trained models for chronic obstructive pulmonary disease motivated by similar reasoning [13]. Detection of physiologic derangements including electrolyte and glucose have been explored as well [14,15]. Interestingly, studies have found success even in disease conditions with less apparent physiologic understanding, such as depression and cirrhosis, which calls for further explainability studies [16,17].

### Equitable algorithm development and implementation

Model performance may or may not be affected by homogenous training data used in the development of DL ECG models. Kaur et al. showed that a model predicting incident heart failure performed significantly worse in younger Black patients. Meanwhile, a similar study by Noseworthy et al,. suggested that race did not impact predictive capability of an ECG-based model predicting reduced ejection fraction [18]. In our study, we demonstrated generalizability of the model to an external test cohort with distinct demographic makeup [7]. This suggests that bias in DL models are dependent not only on the diversity of the training data but also on the specific clinical question being addressed. Given the known risks of bias in medical decision-making, the importance of demonstrating DL’s efficacy across diverse groups is paramount and should become the standard of DL development.

## DISCUSSION

### Clinical implications

DL ECG models create new opportunities to enhance access to diagnostic and prognostic tools, especially in low-resource settings without specialist consultation and advanced imaging studies. These models could provide new screening modalities and reduce diagnostic latency by automated prediction of various cardiac and non-cardiac conditions. Additionally, telemedicine platforms along with ubiquitous consumer devices with biosignal acquisition allow for seamless integration of DL models, allowing for continuous remote monitoring of high-risk populations, particularly in the era of wearable devices. For instance, predictive models could identify asymptomatic individuals at risk of developing atrial fibrillation or heart failure, thus enabling early preventive measures [8]. Meanwhile, the cost-benefit analysis of this new care paradigm remains to be demonstrated in clinical practice.

### Technical and implementation challenges

Despite recent advances, a number of challenges hinder real-world implementation of DL ECG technology. First, there is a relative deficiency of prospective validation studies, which raises concerns about model performance in a variety of clinical, geographic, and environmental contexts. While these models often achieve exceptional performance in single or multi-center cohorts, their generalizability is often limited by data scarcity, highlighting the need for multicenter, multinational datasets. Interpatient variability in chronic conditions poses another barrier, since most models are trained on short-term ECG snapshots rather than longitudinal data collected over time. Furthermore, the uninterpretable nature of DL models results in adoption hesitancy, especially when there is a lack of plausible biological relationship between input and output. This necessitates further explainability research to align model outputs with clinical reasoning. Finally, clinical inertia and a lack of outcomes studies demonstrating real-world efficacy delay adoption, underscoring the urgency to develop pragmatic trials to assess the impact of DL ECG tools on patient outcomes.

### Ethical and equity considerations

The risk of perpetuating healthcare disparities looms large if models are trained on non-representative or homogenous data. It is essential to mitigate those risks by emphasizing community-engaged dataset curation, evaluation of subgroup-specific performance metrics, and exploring strategies such as site-specific fine-tuning to adapt models to local populations [18]. Equitable AI development must prioritize transparency in data sourcing in order to ensure that historical inequities are not inadvertently exacerbated.

### Recent innovations and future directions

Development of novel approaches is underway to address the above challenges while broadening the scope of ECG-based DL applications. For instance, unsupervised learning techniques, such as domain adaptation via adversarial training, reduce reliance on human-labeled datasets-a critical advantage for rare conditions or underrepresented populations [19]. Multimodal architectures integrating ECG with wearable device data (e.g., Photoplethysmography (PPG), ballistocardiography) and point-of-care biomarkers (e.g., continuous glucose monitor) promise richer physiological insights, potentially enabling more comprehensive health assessments. Multi-task learning frameworks, which simultaneously analyze ECG signals for diverse endpoints (e.g., arrhythmia detection and major cardiovascular event prediction), show promise toward more individualized and holistic risk stratification [20]. Future efforts should prioritize collecting longitudinal data to capture temporal dynamics to improve chronic disease tracking. Overall, close collaboration between AI developers and clinicians is pivotal to bridge the gap between algorithmic innovation and patient care.

## CONCLUSION

ECG-based DL models represent a facet of AI-enhanced precision medicine, offering tools for novel diagnostics and innovative care delivery. However, realizing its potential requires keen attention to model generalizability, explainability, and ethical considerations. By addressing these challenges through multidisciplinary collaboration and inclusive design, the field can harness ECG-derived AI to create practice-changing tools for the betterment of our collective health.

## References

[1] Somani S, Russak AJ, Richter F, Zhao S, Vaid A, Chaudhry F, Deep learning and the electrocardiogram: Review of the current state-of-the-art. EP Europace. 2021;23(8):1179–1191. 10.1093/europace/euaa377 PMC8350862 33564873

[2] Al-Alusi MA, Khurshid S, Wang X, Venn RA, Pipilas D, Ashburner JM, Trends in consumer wearable devices with cardiac sensors in a primary care cohort. Circ Cardiovasc Qual Outcomes. 2022;15(7):e008833. 35758032 10.1161/CIRCOUTCOMES.121.008833 PMC9308742

[3] Kaur D, Hughes JW, Rogers AJ, Kang G, Narayan SM, Ashley EA, Race, sex, and age disparities in the performance of ECG deep learning models predicting heart failure. Circ Heart Fail. 2024;17(1):e010879. 38126168 10.1161/CIRCHEARTFAILURE.123.010879 PMC10984643

[4] Bai X, Dong X, Li Y, Liu R, Zhang H. A hybrid deep learning network for automatic diagnosis of cardiac arrhythmia based on 12-lead ECG. Sci Rep. 2024;14(1):24441. 39424921 10.1038/s41598-024-75531-w PMC11489693

[5] Goto S, Solanki D, John JE, Yagi R, Homilius M, Ichihara G, Multinational federated learning approach to train ECG and echocardiogram models for hypertrophic cardiomyopathy detection. Circulation. 2022;146(10):755–769. 35916132 10.1161/CIRCULATIONAHA.121.058696 PMC9439630

[6] Choi J, Lee S, Chang M, Lee Y, Oh GC, Lee HY. Deep learning of ECG waveforms for diagnosis of heart failure with a reduced left ventricular ejection fraction. Sci Rep. 2022;12(1):14235. 35987961 10.1038/s41598-022-18640-8 PMC9392508

[7] Rogers AJ, Bhatia NK, Bandyopadhyay S, Tooley J, Ansari R, Thakkar V, Identification of cardiac wall motion abnormalities in diverse populations by deep learning of the electrocardiogram. NPJ Digit Med. 2025;8(1):21. 39799179 10.1038/s41746-024-01407-y PMC11724909

[8] Yuan N, Duffy G, Dhruva SS, Oesterle A, Pellegrini CN, Theurer J, Deep learning of electrocardiograms in sinus rhythm from US veterans to predict atrial fibrillation. JAMA Cardiol. 2023;8(12):1131–1139. 37851434 10.1001/jamacardio.2023.3701 PMC10585587

[9] Al-Zaiti SS, Martin-Gill C, Zègre-Hemsey JK, Bouzid Z, Faramand Z, Alrawashdeh MO, Machine learning for ECG diagnosis and risk stratification of occlusion myocardial infarction. Nat Med. 2023;29(7):1804–1813. 37386246 10.1038/s41591-023-02396-3 PMC10353937

[10] Liang C, Yang F, Huang X, Zhang L, Wang Y. Deep learning assists early-detection of hypertension-mediated heart change on ECG signals. Hypertens Res. 2025;48(2):681–692. 39394520 10.1038/s41440-024-01938-7

[11] Lee E, Ito S, Miranda WR, Lopez-Jimenez F, Kane GC, Asirvatham SJ, Artificial intelligence-enabled ECG for left ventricular diastolic function and filling pressure. NPJ Digit Med. 2024;7(1):4. 38182738 10.1038/s41746-023-00993-7 PMC10770308

[12] Butler L, Gunturkun F, Chinthala L, Karabayir I, Tootooni MS, Bakir-Batu B, AI-based preeclampsia detection and prediction with electrocardiogram data. Front Cardiovasc Med. 2024;11:1360238. 38500752 10.3389/fcvm.2024.1360238 PMC10945012

[13] Moran I, Altilar DT, Ucar MK, Bilgin C, Bozkurt MR. Deep transfer learning for chronic obstructive pulmonary disease detection utilizing electrocardiogram signals. IEEE Access. 2023;11:40629–40644.

[14] Porumb M, Stranges S, Pescape A, Pecchia L. Precision medicine and artificial intelligence: A pilot study on deep learning for hypoglycemic events detection based on ECG. Sci Rep. 2020;10(1):170. 31932608 10.1038/s41598-019-56927-5 PMC6957484

[15] Galloway CD, Valys AV, Shreibati JB, Treiman DL, Petterson FL, Gundotra VP, Development and validation of a deep-learning model to screen for hyperkalemia from the electrocardiogram. JAMA Cardiol. 2019;4(5):428–436. 30942845 10.1001/jamacardio.2019.0640 PMC6537816

[16] Habib A, Vaniya SN, Khandoker A, Karmakar C. MDDBranchNet: A Deep Learning Model for Detecting Major Depressive Disorder Using ECG Signal. IEEE J Biomed Health Inform. 2024;28(7):3798–3809. 38954560 10.1109/JBHI.2024.3390847

[17] Ahn JC, Attia ZI, Rattan P, Mullan AF, Buryska S, Allen AM, Development of the AI-cirrhosis-ECG score: An electrocardiogram-based deep learning model in cirrhosis. Am J Gastroenterol. 2022;117(3):424–432. 35029163 10.14309/ajg.0000000000001617 PMC9727935

[18] Noseworthy PA, Attia ZI, Brewer LC, Hayes SN, Yao X, Kapa S, Assessing and mitigating bias in medical artificial intelligence: The effects of race and ethnicity on a deep learning model for ECG analysis. Circ Arrhythm Electrophysiol. 2020;13(3):e007988. 32064914 10.1161/CIRCEP.119.007988 PMC7158877

[19] Chen M, Wang G, Chen H, Ding Z. Adaptive region aggregation network: Unsupervised domain adaptation with adversarial training for ECG delineation. ICASSP. 2020:1274–1278.

[20] Lin CH, Liu ZY, Chu PH, Chen JS, Wu HH, Wen MS, A multitask deep learning model utilizing electrocardiograms for major cardiovascular adverse events prediction. NPJ Digit Med. 2025;8(1):1. 39747648 10.1038/s41746-024-01410-3 PMC11696183

